# The *Rhizophagus irregularis* permease RiFTR1 functions without a ferroxidase partner for reductive iron transport

**DOI:** 10.1038/s41598-025-88416-3

**Published:** 2025-02-18

**Authors:** Elisabeth Tamayo, Víctor Manuel López-Lorca, Chaeeun Shim, Olga López-Castillo, Araceli G. Castillo, Natalia Requena, J. Philipp Benz, Nuria Ferrol

**Affiliations:** 1 Departamento de Microbiología del Suelo y Sistemas Simbióticos, Estación Experimental del Zaidín, CSIC, Granada, Spain; 2 Holzforschung München, TUM School of Life Sciences, Technische Universität München, Freising, Germany; 3 Department of Biochemical Engineering, Bernard Katz Building, University College London, London, UK; 4 Instituto de Hortofruticultura Subtropical y Mediterránea “La Mayora”, Universidad de Málaga-CSIC (IHSM, UMA-CSIC), Málaga, Spain; 5 Molecular Phytopathology, Botanical Institute, Karlsruhe Institute of Technology (KIT), Karlsruhe, Germany

**Keywords:** *Rhizophagus irregularis*, High-affinity reductive pathway, Ftr1 iron transporter, Ferroxidases, Arbuscular mycorrhizal symbiosis, Microbiology, Plant sciences

## Abstract

The contribution of arbuscular mycorrhizal fungi (AM fungi) to plant iron (Fe) acquisition has been demonstrated in several studies. A previous investigation revealed that the AM fungus *Rhizophagus irregularis* utilizes a high-affinity reductive pathway for Fe uptake, mediated by the Fe transporter RiFTR1. In this study, we used a genome-wide approach in *R. irregularis* to find genes encoding ferroxidases of the multicopper oxidase (MCO) gene family in an attempt to identify the ferroxidase partner of RiFTR1. Nine genes putatively encoding MCOs (*RiMCO1-9*) were identified. Yeast complementation assays demonstrated that RiMCO1 and RiMCO3 can function as ferroxidases, suggesting their involvement in the reductive Fe uptake pathway. Surprisingly, RiFTR1 was capable of transporting Fe in yeast without a ferroxidase partner, resembling the Fe transport mechanism of plant IRT1-like systems. *RiFTR1* exhibited increase expression in arbuscules. Overexpression of *RiFTR1* in *Medicago truncatula* roots led to enhanced mycorrhizal colonization and arbuscule abundance, highlighting the significance of Fe for AM symbiosis.

**Supplementary Information:**

The online version contains supplementary material available at 10.1038/s41598-025-88416-3.

## Introduction

Iron (Fe) is an essential metal element, however, it can become extremely toxic at high concentrations^1^. Therefore, its biological levels are finely regulated in living cells of all organisms, which have evolved sophisticated tools to maintain Fe homeostasis^2–4^. At the same time, although Fe is abundant in nature, its bioavailability is very limited owing to its oxidation into insoluble ferric hydroxides by atmospheric oxygen^5^. For this reason, high-affinity Fe transport systems are required for its uptake.

The symbiosis with arbuscular mycorrhizal (AM) fungi belonging to the Glomeromycotina (Mucoromycota phylum) is an evolutionary ancient strategy of plants to increase their nutrient supply^6^. AM fungi are obligate biotrophs that form an extensive external mycelium in the soil that can absorb nutrients beyond the depletion zone that develops around the roots^7–9^. These nutrients are then transferred to the host plant in the colonized cortical root cells, where the fungus forms highly branched and specialized structures called arbuscules^10^.

AM fungi provide, therefore, an additional pathway for the uptake and transport of low mobility nutrients in the soil, particularly phosphorus and nitrogen, but also micronutrients such as Fe, copper and zinc^6,11–13^. In return, the plants provide fixed carbon in the form of sugars and lipids to their symbiotic fungi^14–17^. The contribution of AM fungi to plant Fe uptake has been shown in several studies^18–20^.

Iron uptake strategies in plants and fungi are adapted to their environments and physiological needs. Plants have evolved two distinct Fe acquisition strategies: Strategy I and Strategy II. Strategy I, used by non-grass species, involves the reduction of Fe(III) to Fe(II) by ferric chelate reductase enzymes at the root surface, followed by the transport of Fe(II) into root cells via specific transporters such as IRT1 (Iron-Regulated Transporter 1). Strategy II, employed by grasses, involves the secretion of phytosiderophores that chelate Fe(III) in the soil and form soluble complexes that are then transported into root cells via specialized transporters like YS1 (Yellow Stripe 1). On the other hand, fungi often utilize siderophores, high-affinity Fe-chelating compounds, secreted into the environment to bind Fe(III) and transport it back into the cell via specific siderophore transporters^21^. Some fungi also possess reductive iron uptake systems similar to Strategy I in plants, where Fe(III) is first reduced to Fe(II)by membrane-bound ferrireductases. Fe(II) is then rapidly internalized by the concerted action of a ferroxidase (FET3) and an iron permease (FTR1) that form a plasma membrane protein complex^22–24^. Fe(II) is first oxidized by FET3, and then transported into the cytosol as Fe(III) by FTR1 via a channeling mechanism^25^. The Fet3-Ftr1 high affinity reductive Fe uptake system has been found encoded in almost all archived fungal genomes^26,27^. Fungal ferroxidases are plasma membrane proteins with a single transmembrane domain and an extracellular multicopper oxidase domain responsible for the ferroxidase activity that belong to the widely distributed family of multicopper oxidase (MCO) proteins^28^. Other members of this family are laccases, ascorbate oxidases, bilirubin oxidases and potential ferroxidases/laccases exhibiting either one or both enzymatic functions^29,30^. Fungal Fet3-like ferroxidases have been characterized in many fungi including *Pichia pastoris*, *Fusarium graminearum* and *Candida albicans*^31–33^. Homologous of the *S. cerevisiae* Fe permease FTR1 have also been identified in various fungi, including *Schizosaccharomyces pombe*, the plant endophytic fungus *Piriformospora indica* and the arbuscular mycorrhizal fungus *Rhizophagus irregularis*^34–37^. We could demonstrate that the *R. irregularis RiFTR1*gene encodes a plasma membrane Fe uptake transporter that is preferentially induced inside the plant as compared with the extraradical mycelium^36^, suggesting a role in maintaining the Fe homeostasis within colonized roots. However, the ferroxidase partner of the plasma membrane Fe transporter RiFTR1 of *R. irregularis* remains unidentified and the significance of RiFTR1 in the AM symbiosis needs to be unraveled. With the aim of further characterizing RiFTR1, here we have used a genome-wide approach to identify its ferroxidase partner and we have functionally characterized RiFTR1 using a host-induced overexpression approach.

## Results

### *R. irregularis* has at least nine multicopper oxidases in its genome

As a first step to identify the ferroxidase partner of RiFTR1, a search for putative MCOs was carried out in the *R. irregularis* genome databases (*R. irregularis* DAOM 181602, A1, B3, C2, A4, A5 and DAOM 197198). Nine genes putatively encoding MCOs were identified (*RiMCO*1-*RiMCO9*) and the cDNA sequences of RiMCO1, RiMCO3, RiMCO4, RiMCO5 and RiMCO6 were experimentally confirmed by RACE using cDNAs from extraradical mycelium (ERM). *RiMCO2*, *RiMCO7*, *RiMCO8* and *RiMCO9* could not be cloned because no band could be obtained in the RACE experiments probably because they are expressed in other fungal structures. The length of the predicted RiMCO proteins ranges from 517 to 708 amino acids (Table S3). Sequence identities among the deduced amino acid sequences of RiMCO1-9 ranges from 24 to 82%, with RiMCO6 having the lowest identity to the others (≤ 29%) (Fig. S1A). RiMCO4 and RiMCO7 show the highest degree of identity (82%). Relationships between the nine *R. irregularis* MCOs are also reflected in the phylogenetic tree based on their amino acid similarities (Fig. S1B).

**Fig. 1:**
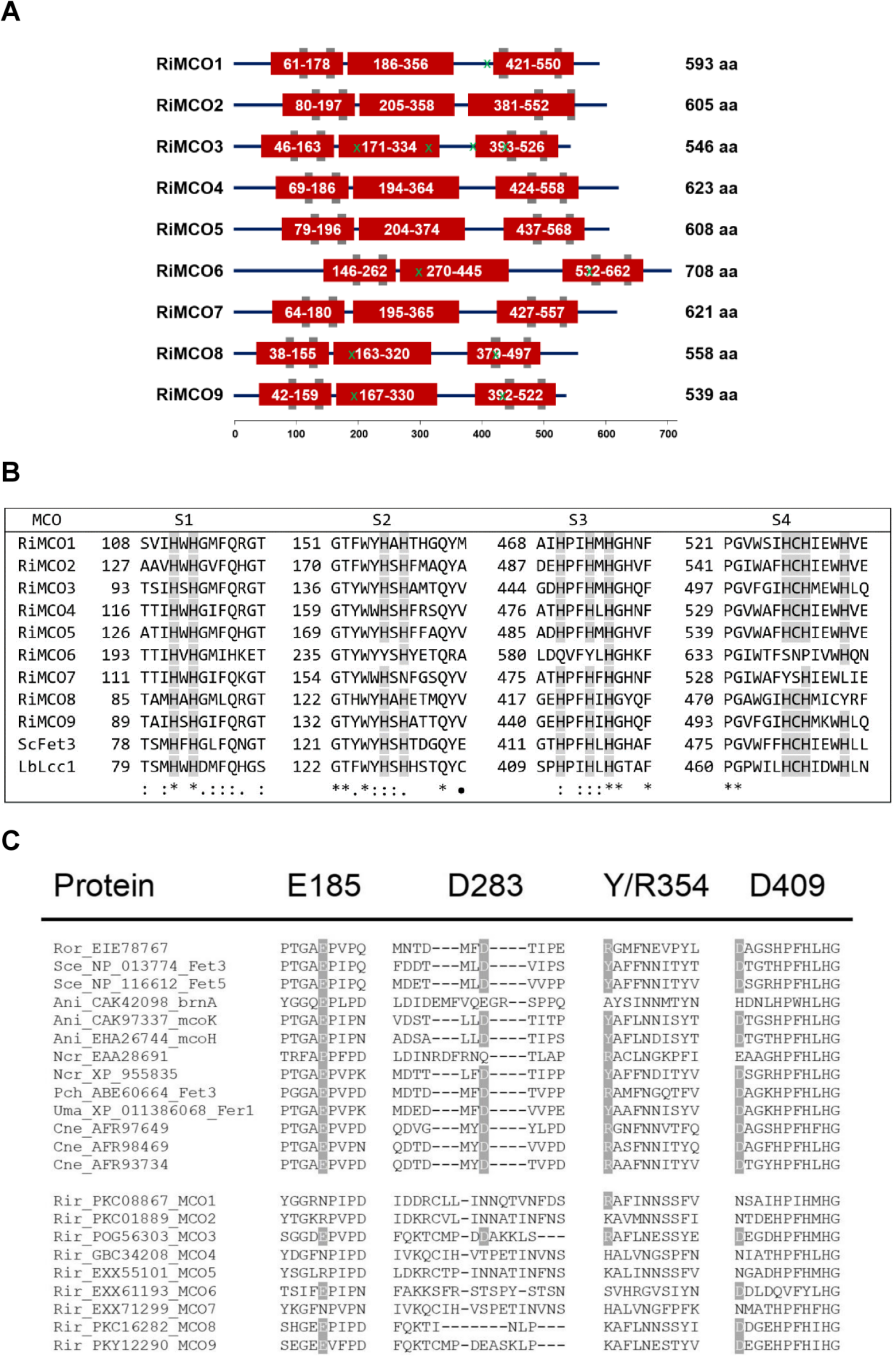
**(A) Domain organization in the *Rhizophagus irregularis *MCO proteins.** Domains were identified by SMART analyses. Red boxes indicate the cupredoxin-like domains. Grey boxes represent signature motifs S1 to S4. Numbers represent amino acid positions. Protein lengths in amino acids (aa) are indicated on the right. Residues presumably essential for Fe oxidation are indicated with green crosses. **(B) Sequence alignment of the four copper-binding sites (signature motifs S1 to S4) of the nine *Rhizophagus irregularis *MCOs**,** the *Saccharomyces cerevisiae *ferroxidase Fet3 and the *Laccaria bicolor *laccase Lcc1.** Histidine (H) and cysteine (C) copper ligands are indicated. [The conserved 10 histidines and the cysteine residue involved in the coordination of copper are shown]. A black circle denotes a residue in signature sequence S2 that is always a cysteine (C) in classical laccases, but not in Fet3 ferroxidases. **(C) Alignment of the four regions known to function in oxidation of Fe(II) to Fe(III) in *S. cerevisiae *Fet3 with the corresponding sequence regions of Fet3-type ferroxidases from fungi and Rhizophagus irregularis MCOs.** The upper block presents the cluster of Fet3-like enzymes and the lower block the cluster of *R. irregularis* ferroxidases/laccases. The residues involved in ferroxidase activity (E185, D283, Y/R354 and D409) are highlighted in grey. Organisms: Ani, *Aspergillus niger*; Cne, *Cryptococcus neoformans*; Ncr, *Neurospora crassa*; Pch, *Phanerochaete chrysosporium*; Rir, *Rhizophagus irregularis*; Ror, *Rhizopus oryzae*; Sce, *Saccharomyces cerevisiae*; Uma, *Ustilago maydis*. Protein NCBI identification numbers are indicated.

The nine RiMCOs contain three cupredoxin-like domains linked by external interdomains (Fig. 1A). Alignment of the deduced amino acid sequences of RiMCO1-9 with other fungal MCOs revealed that the nine RiMCOs have the four conserved regions that contain the residues involved in copper coordination, a characteristic signature of all MCOs (Fig. 1B). RiMCO1, RiMCO3, RiMCO6, RiMCO8 and RiMCO9 have at least one of the four residues (E185, D283, Y354 and D409) that were shown in *S. cerevisiae *to be essential for Fe oxidation^38,39^ (Fig. 1A and C), a characteristic of ferroxidases and ferroxidases/laccases with a main ferroxidase activity^40^. RiMCO1, RiMCO3, RiMCO6 and RiMCO9 are predicted to have an N-terminal transmembrane domain, but lack the C-terminal transmembrane domain typical of ferroxidases. RiMCO8 was predicted to have three transmembrane domains, one at the N-terminus and the other two at the C-terminus (Table S3).

**Fig. 2:**
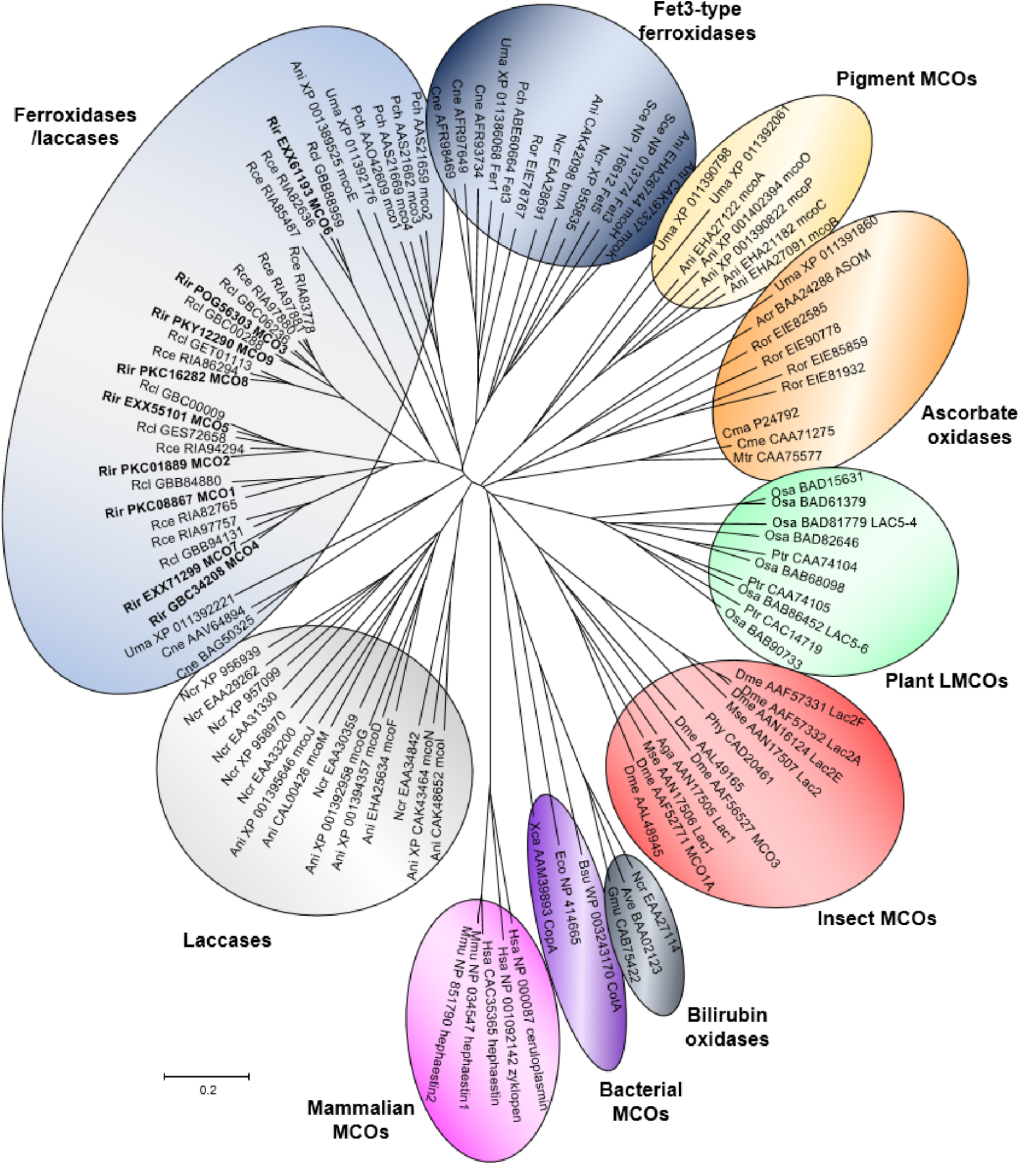
**Unrooted Neighbor-Joining tree of the MCO gene family.** Organisms: Acr, *Acremonium* sp.; Aga, *Anopheles gambiae*; Ani, *Aspergillus niger*; Ave, *Albifimbria verrucaria*; Bsu, *Bacillus subtilis*; Cma, *Cucurbita maxima*; Cme, *Cucumis melo*; Cne, *Cryptococcus neoformans*; Dme, *Drosophila melanogaster*; Eco, *Escherichia coli*; Gmu, *Gliomastix murorum*; Hsa, *Homo sapiens*; Mmu, *Mus musculus*; Mse, *Manduca sexta*; Mtr, *Medicago truncatula*; Ncr, *Neurospora crassa*; Osa, *Oryza sativa*; Pch, *Phanerochaete chrysosporium*; Phy, *Pimpla hypochondriaca*; Ptr, *Populus trichocarpa*; Rce, *Rhizophagus cerebriforme*; Rcl, *Rhizophagus clarus*; Rir, *Rhizophagus irregularis*; Ror, *Rhizopus oryzae*; Sce, *Saccharomyces cerevisiae*; Uma, *Ustilgo maydis*; Xca, *Xanthomonas campestris*. *Rhizophagus irregularis* MCOs are emphasized in bold. Protein NCBI identification numbers are indicated.

A phylogenetic analysis of MCO sequences of different taxonomic groups revealed that all RiMCOs clustered together in the ferroxidase/laccase group (Fig. 2). In contrast to the MCOs of the other fungi used in the phylogenetic analysis, none of the RiMCOs clustered with the Fet3-type ferroxidases. The same is true also for the AM fungi *Rhizophagus cerebriforme* and *Rhizophagus clarus*, both belonging to the Glomeromycotina subphylum^41^.

These in silico analyses showed that although there is no homologue to Fet3-type ferroxidases *sensu stricto* in the *R. irregularis* genome, there are nevertheless several potential candidates that could be the ferroxidase partners of the Fe transporters: RiMCO1, RiMCO3, RiMCO6, RiMCO8 and RiMCO9, having at least one of the four residues essential for Fe oxidation and a predicted transmembrane domain.

### RiMCOs are differentially expressed in the intraradical and extraradical mycelium

**Fig. 3:**
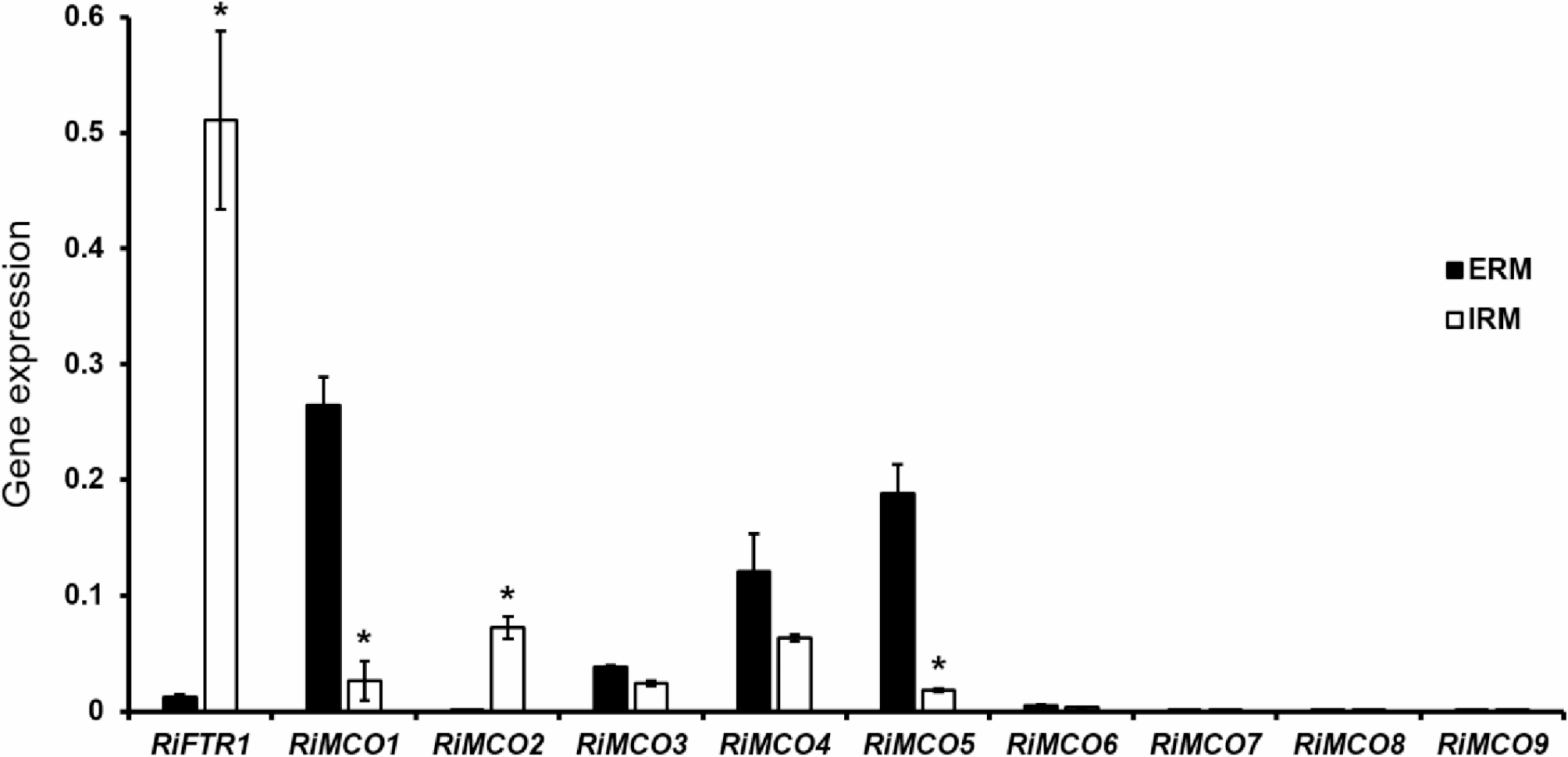
**Relative expression of the Fe transporter *RiFTR1  *and the *MCO*** **genes in extraradical mycelia (ERM) and intraradical mycelia (IRM) of *Rhizophagus irregularis***. *RiFRT1* and *RiMCO* gene expression was assessed in ERM developed in monoxenic cultures (ERM) and *R. irregularis*-colonized carrot roots grown in monoxenic cultures and lacking ERM (IRM). Samples were normalized using the housekeeping gene *RiTEF*. Relative expression levels were calculated by the 2^−ΔCT^ method. Data are means +/- standard error. Asterisks show statistically significant differences (*p* < 0.05) relative to the ERM, according to the Fisher’s LSD test.

To further characterize the RiMCOs, we decided to compare *RiMCOs* gene expression in the intraradical mycelium (IRM) and ERM under control conditions. Quantitative gene expression analysis was performed by real-time RT-PCR on ERM collected from the hyphal compartment of *R. irregularis* liquid monoxenic cultures (Fig. S2B) and on carrot mycorrhizal roots (20% mycorrhizal colonization) lacking ERM (Fig. S2C) that were grown for two weeks in a densely colonized hyphal compartment of split Petri dishes. In the ERM, *RiMCO1*,* RiMOC4* and *RiMCO5* were the most highly expressed genes, followed by *RiMCO3*. *RiMCO6* expression was barely detected in the ERM (Fig. 3). However, *RiMCO2*, whose encoded protein lacks the signatures typical of ferroxidases, was the most highly expressed gene in the IRM. Transcript levels of *RiMCO1* and *RiMCO5* were approximately 10-fold higher in the ERM than in the IRM of the monoxenically grown carrot roots. No significant differences were observed between the expression levels of *RiMCO3*, *RiMCO4* and *RiMCO6* in both fungal structures. *RiMCO7*, *RiMCO8* and *RiMCO9* were not expressed or were below the detection limit in both fungal structures (Fig. 3). The expression of none of the genes encoding proteins with the signatures typical of ferroxidases, namely RiMCO1, RiMCO3, RiMCO6, RiMCO8 and RiMCO9, paralleled the expression of the Fe transporter *RiFTR1*, which was more highly expressed in the IRM.

### Fe regulates *RiMCO1*, *RiMCO3* and *RiMCO4* gene expression

**Fig. 4:**
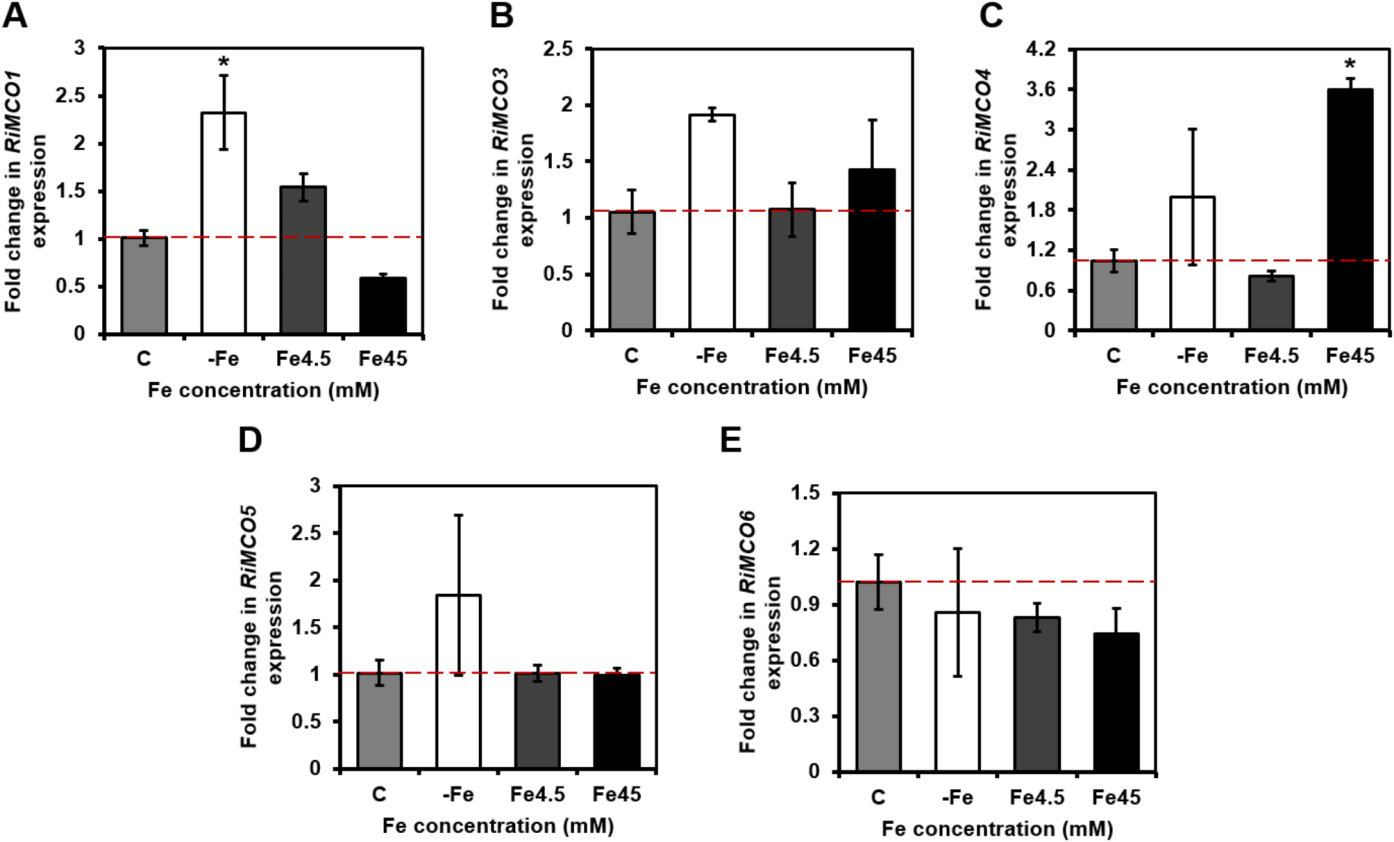
**Effect of iron on the expression of the *Rhizophagus irregularis MCO*** **genes.*** R. irregularis* was grown for 2 weeks in M-C media containing 0.045 mM Fe (control, C), 4.5 mM Fe (Fe4.5) or 45 mM Fe (Fe45), or in M-C without Fe and exposed for 3 days to ferrozine (-Fe). *RiMCO1* (A), *RiMCO3* (B), *RiMCO4* (C), *RiMCO5* (D) and *RiMCO6* (E) gene expression. Data were normalized using the housekeeping gene *RiTEF*. Relative expression levels were calculated by the 2^−ΔΔCT^ method. Data are means +/- standard error. Asterisks show statistically significant differences (*p* < 0.05) compared to the control value, according to the Tukey’s (normal distribution) or Dunn’s (no normal distribution) test.

Since fungal ferroxidases are transcriptionally regulated by Fe^4^, in an attempt to identify the potential *R. irregularis* ferroxidases that interact with the Fe permease RiFTR1, *RiMCOs* transcript levels were analyzed in ERM grown in the presence of different Fe concentrations (Fig. 4). Relative to the ERM grown in control M media containing 0.045 mM Fe, *RiMCO1* and *RiMCO3* gene expression was observed to be 2.3- (*p*-value < 0.05) and 1.8-fold up-regulated (*p*-value = 0.089), respectively, when the ERM was grown in medium lacking Fe and exposed to the Fe chelator ferrozine for 3 d. On the other hand, development of the fungus in the presence of 45 mM Fe induced a slight albeit not statistically significant down-regulation of *RiMCO1* expression, and a 3.5-fold up-regulation of *RiMCO4* expression. Transcript levels of *RiMCO5* and *RiMCO6* were not significantly affected by the amount of Fe present in the culture medium. *RiMCO2*, *RiMCO7*, *RiMCO8* and *RiMCO9* were not expressed in our experimental conditions or were below the detection limit. *RiMCO1* and *RiMCO3* were the only *MCO *genes displaying a gene expression pattern typical of a high-affinity Fe transport system^22^, suggesting that RiMCO1 and RiMCO3 might be the partners of the Fe permease RiFTR1. Moreover, *RiMCO4* was up-regulated in ERM grown in media supplemented with 45 mM Fe.

### RiFTR1 can take up fe without the involvement of a ferroxidase partner in yeast

**Fig. 5:**
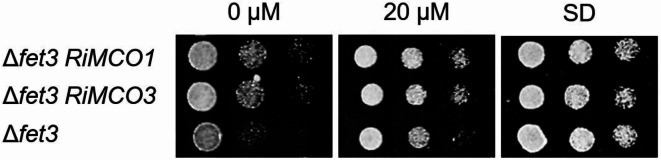
**Analysis of the** **in vivo** **role of RiMCO1 and RiMCO3 in iron transport in yeast.** Δ*fet3* cells untransformed or expressing *RiMCO1* or *RiMCO3* were examined for growth under Fe-limiting conditions (0 and 20 µM Fe). Control growth was observed in SD plates.

Following the observation that the coding sequences of *RiMCO1* and *RiMCO3* present the functional domains of ferroxidases and that their transcript levels increase under Fe-limiting conditions, we next examined whether they have ferroxidase activity and could act together with the plasma membrane Fe transporter RiFTR1 in the reductive Fe uptake pathway of *R. irregularis*^36^. To that end, we first assessed if both proteins can restore the inability of a *S. cerevisiae* mutant lacking Fet3 ferroxidase activity (Δ*fet3*) to grow under Fe-limiting conditions. For this purpose, the *RiMCO1* and *RiMCO3* full length cDNAs were cloned into a yeast expression multicassette and the capability of the *RiMCO1*- and *RiMOC3*-expressing yeast mutants to grow under these conditions was determined. Indeed, relative to the untransformed Δ*fet3* cells, the *RiMCO1*- and *RiMCO3*-expressing Δ*fet3* cells improved yeast growth under Fe-limiting conditions (Fig. 5), suggesting that RiMCO1 and RiMCO3 can oxidize ferrous iron to ferric iron and assemble to the yeast Ftr1 permease to enable the mutant to take up iron.

**Fig. 6:**
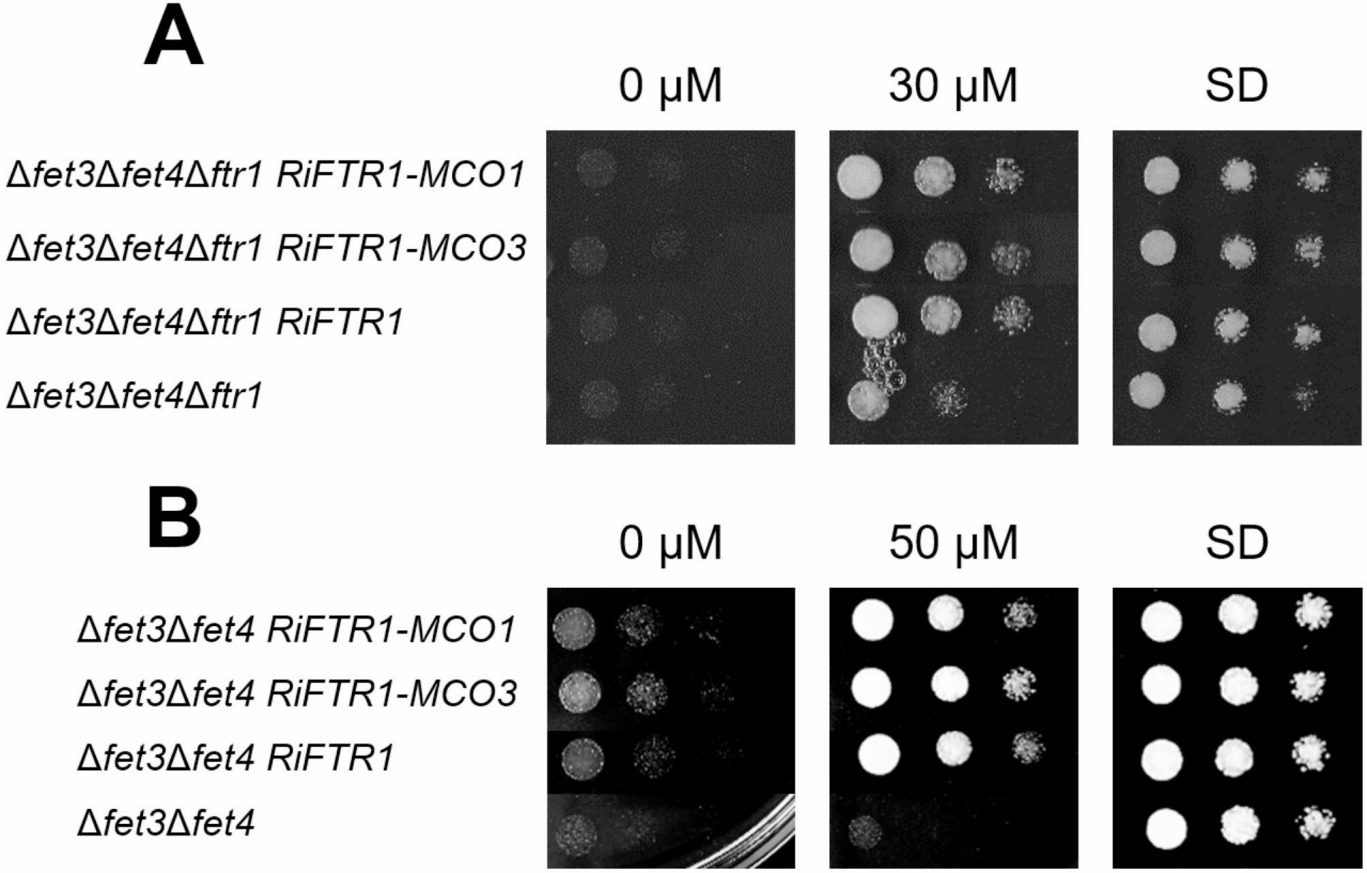
**Analysis of the in vivo role of RiFTR1 and RiMCOs in iron transport in yeast mutant strains lacking Fet3 and Fet4.** Δ*fet3*Δ*fet4*Δ*ftr1***(A)** and Δ*fet3*Δ*fet4***(B)** yeast mutant strains were transformed with different constructs in order to search for the partner of the Fe transporter FTR1.

**Fig. 7:**
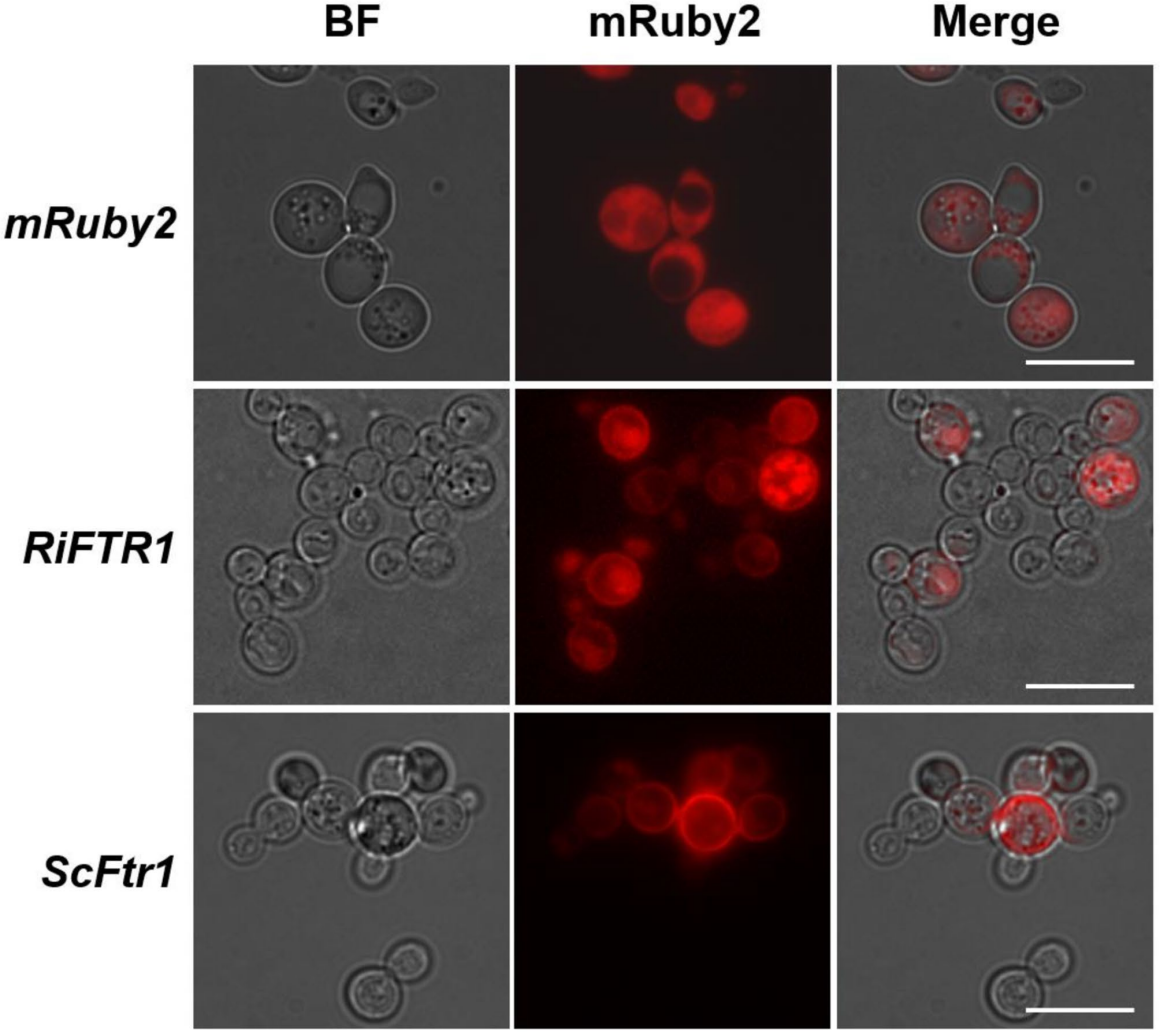
**Analysis of the in vivo localization of RiFTR1 in a high-affinity reductive Fe pathway yeast mutant.** Δ*fet3*Δ*fet4* cells transformed with a vector expressing *mRuby2* (first row), *RiFTR1-mRuby2* (second row) or *ScFtr1-mRuby2* (third row) were visualized with a Zeiss Axio fluorescent microscope. BF, bright field (first column); mRuby2, red channel (second column); Merge, combination of BF and red channels (third column).

We next examined if RiMCO1 and RiMCO3 could be the ferroxidase partners of the *R. irregularis* RiFTR1. To that end, the Δ*fet3*Δ*fet4*Δ*ftr1* mutant strain of *S. cerevisiae* lacking the ferroxidase Fet3 and the low-affinity and high-affinity Fe permeases Fet4 and Ftr1, respectively, was transformed with the empty vector (negative control) or with plasmids containing *RiFTR1*-*R*i*MCO1*,* RiFTR1*-*RiMCO3* or *RiFTR1*. The triple mutant is unable to grow in Fe-limited (0 and 30 µM Fe) media. Surprisingly, in media supplemented with 30 µM Fe, growth was restored in the yeast cells expressing any of the three constructs (Fig. 6A). Furthermore, co-expression of RiMCO1 or RiMCO3 with RiFTR1 did not help to improve further the yeast growth under Fe-limiting conditions. To confirm these results, the Fe uptake-deficient Δ*fet3*Δ*fet4* double mutant strain of *S. cerevisiae* was transformed with the same plasmids. As expected, expression of *RiFTR1* complemented the mutant phenotype and there was no difference between the strains co-expressing *RiFTR1* alone or in combination with any of the *MCO* genes (Fig. 6B). Localization analysis of the RiFTR1-mRuby2 fusion protein revealed a clear fluorescent signal at the cell periphery indicative of a plasma membrane localization. Additionally, RiFTR1-mRuby2 fusion protein was observed within the perinuclear endoplasmic reticulum membrane, a phenomenon commonly found in yeast membrane protein overexpression assays (Fig. 7). The plasma membrane localization of RiFTR1 suggests that it is sufficient for the uptake of Fe under Fe-limiting conditions in yeast, without the need for the joint action of a ferroxidase partner. Therefore, the mechanism of Fe transport by RiFTR1 appears to be more similar to that of the plant IRT1 Fe transporters, which do not require a multicopper oxidase for iron transport^42^ than to the common mechanism of other Ftr1 iron transporters in fungi.

### *RiFTR1* is mainly expressed in the arbuscule-containing cells and its overexpression promotes arbuscule development

**Fig. 8:**
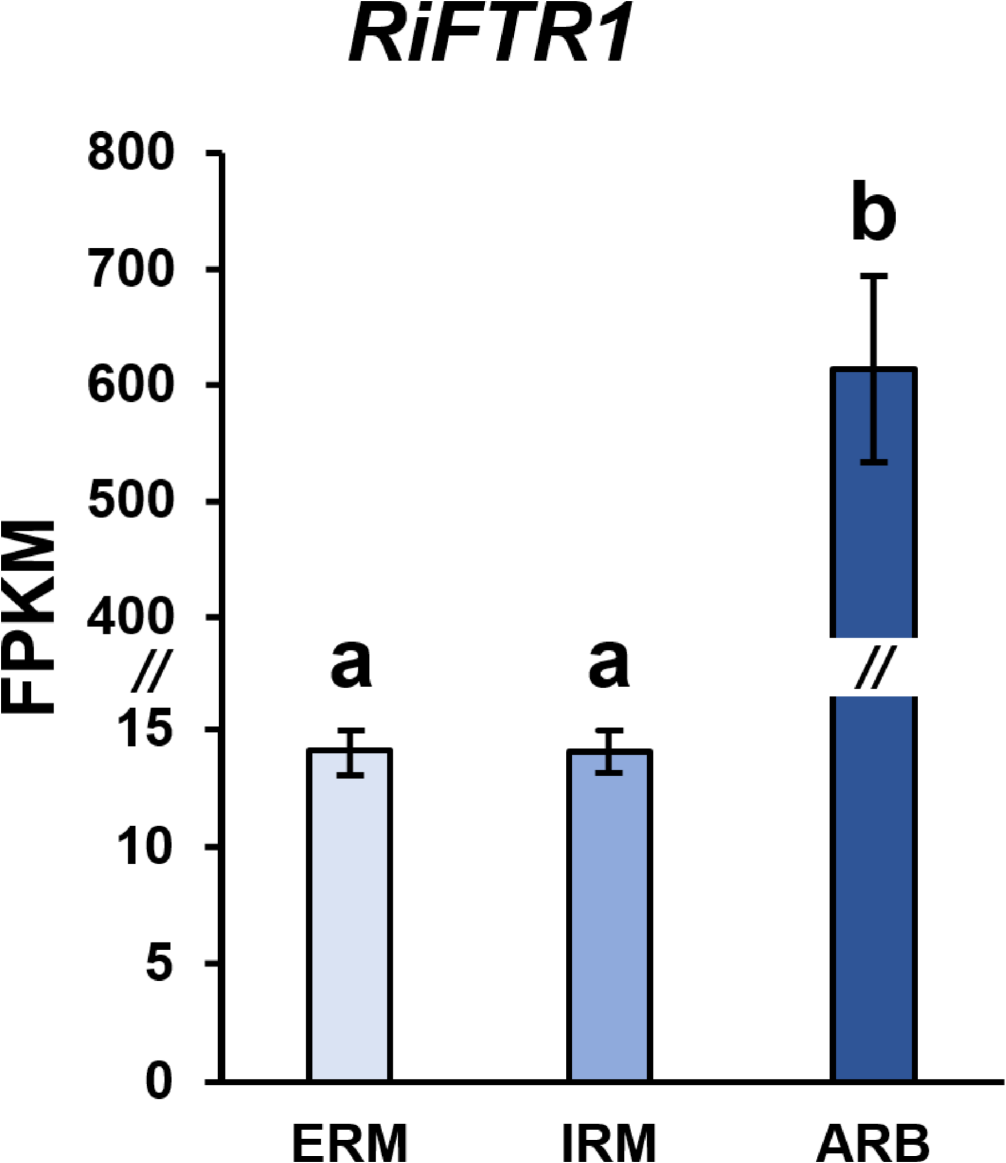
**Expression patterns of *RiFTR1*** **in extraradical mycelia (ERM), intraradical hyphae (IRM) and arbuscule-containing cells (ARB) of *Rhizophagus irregularis***. *RiFTR1* gene expression based on RNAseq analyses of ERM grown in monoxenic cultures as well as IRM and ARB collected by laser microdissection, from *Medicago truncatula* mycorrhizal roots. Data are means +/- standard error. Different letters indicate statistically significant differences (*p* < 0.05).

It was previously shown that *RiFTR1* is expressed to a greater extent in mycorrhizal roots than in ERM^36^. To gain further insight into its precise localization, *RiFTR1* expression levels were investigated using in silico analysis of RNASeq data generated from ERM grown in monoxenic cultures and from laser microdissected *Medicago truncatula *root cells containing intraradical hyphae or arbuscules^43,44^. Although *RiFTR1* transcripts were detected in all fungal structures analyzed, a 43-fold increase in expression was observed in root cells containing arbuscules relative to expression in intraradical hyphae and ERM (Fig. 8), indicating that RiFTR1 could play a role in maintaining Fe homeostasis during arbuscule development and/or function.

**Fig. 9:**
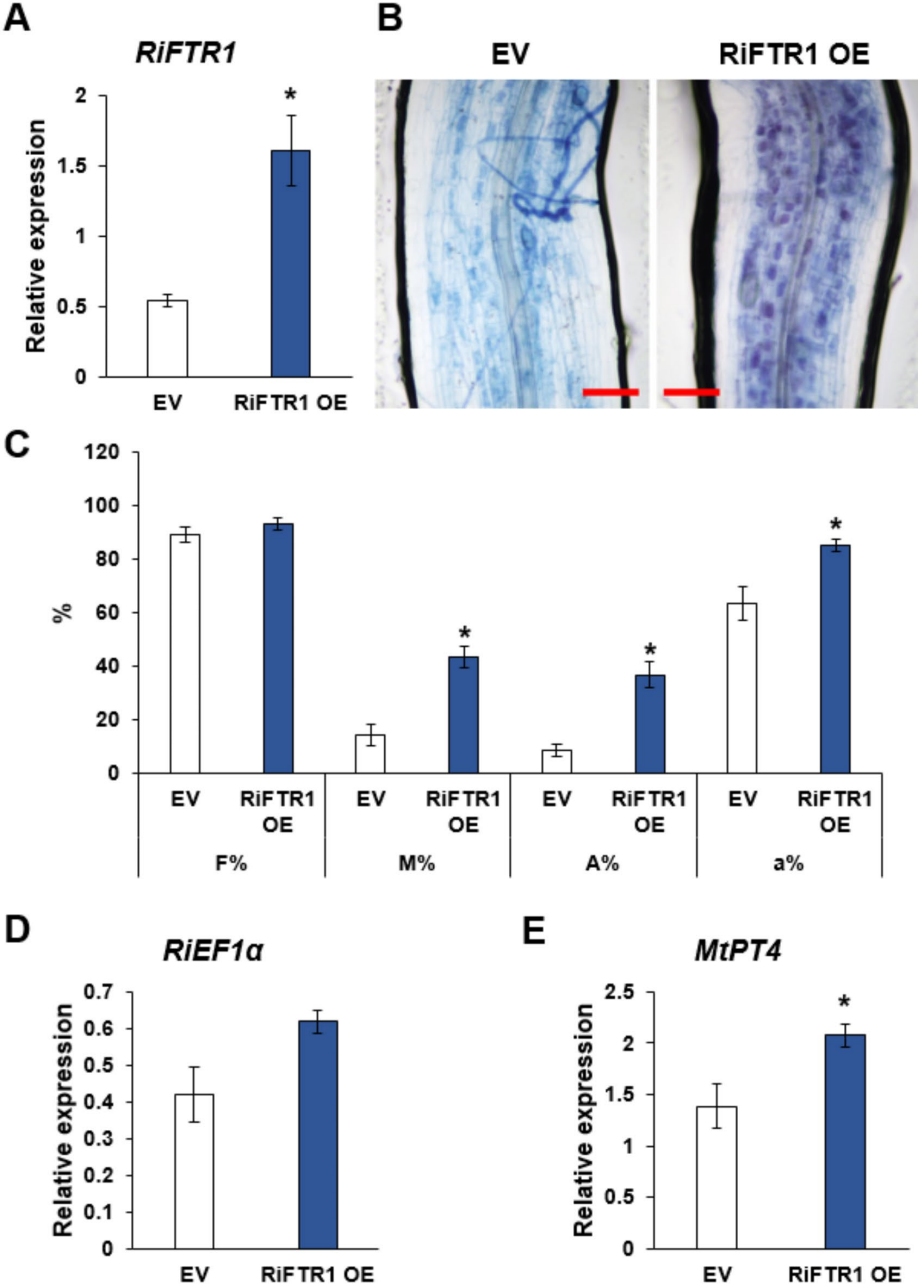
**Overexpression of *RiFTR1 *in mycorrhizal *Medicago truncatula***. **(A)** Ectopic expression of *RiFTR1* in mycorrhizal roots of *M. truncatula* composite plants transformed with and empty vector (EV) compared with *RiFTR1*-expressing plants (RiFTR1 OE). **(B)** Representative pictures of the observed colonization in *M. truncatula* roots stained with trypan blue (blue signal). Scale bars are 100 µM. **(C)** Quantification of *R. irregularis* colonization by the Trouvelot method. F% represents the frequency of mycorrhization, M% the intensity of colonization, A% the relative abundance of arbuscules, and a% the absolute abundance of arbuscules or arbuscule abundance in mycorrhizal root fragments. **(D)** Transcript levels of *RiEF1*α in roots of control EV plants compared with RiFTR1 OE plants. **(E)** Transcript levels of the *M. truncatula* symbiotic marker *MtPT4* in roots of EV plants compared with RiFTR1 OE plants. Relative expression levels were calculated by the 2^−ΔCT^ method. Data are means +/- standard errors (*n* = 4). Statistical significance is indicated with asterisks (*p > 0.05).

In order to link the function of RiFTR1 to the development of the AM symbiosis, and, since there are no known stable transformation protocols for AM fungi, we attempted its inactivation using HIGS^14,45^. Despite several attempts using two different RNAi-silencing constructs, the expression of *RiFTR1* could not be successfully knocked down (data not shown). As an alternative approach to investigate the function of RiFTR1, we ectopically overexpressed it in *M. truncatula* roots and carried out mycorrhizal colonization assays. To that end, the open reading frame of *RiFTR1* was expressed in hairy roots of *M. truncatula* under the control of the constitutive *Ubiquitin1* promoter. After screening the transformed *M. truncatula* roots by red fluorescence, they were inoculated with *R. irregularis*. *RiFTR1* overexpression was confirmed by RT-qPCR in mycorrhizal roots of the composite plants. A 2.9-fold increase in *RiFTR1* expression was detected in the *RiFTR1*-overexpressing plants (RiFTR1 OE) compared to those transformed with the empty vector (Fig. 9A). Interestingly, plants overexpressing *RiFTR1* in roots were more colonized by *R. irregularis* and had 4 times more arbuscules than EV plants (Fig. 9B and C), and this was not due to a higher root or plant weight (Fig. S3). The higher mycorrhizal intensity of RiFTR1 OE plants paralleled to a higher expression of the symbiotic phosphate transporter *MtPT4*, but not to the expression of the fungal translation elongation factor *RiEFα* (Fig. 9D and E), suggesting that Fe homeostasis could be particularly important during arbuscule development or function and that RiFTR1 could play an important role in this process.

## Discussion

Iron is an essential micronutrient for life that plays a central role in plant-microbe interactions^46,47^. Previous observations that the plasma membrane Fe transporter RiFTR1 of the AM fungus *R. irregularis* is highly expressed during the *in planta*phase of the fungus pointed towards a central role of this transporter in the AM symbiosis^36^. Here, we report that RiFTR1, unlike its fungal homologs, does not require a multicopper oxidase for its function and that plant host induced overexpression of *RiFTR1* promotes mycorrhizal colonization.

Our search for potential ferroxidase partners for the Fe permease RiFTR1 indicated that the genome of *R. irregularis* harbors nine sequences putatively encoding MCOs. In silico protein analyses showed that the putative RiMCOs contain the three cupredoxin-like domains typical of multicopper oxidases^35^. Phylogenetic analysis of the RiMCO protein sequences revealed that they group together with the orthologous sequences of other AM fungi within the ferroxidase/laccase branch of fungal MCOs, which indicated that *R. irregularis* does not have any ferroxidase *sensu stricto*. To date, ferroxidase genes (coding Fet3-type ferroxidases or ferroxidases/laccases) have been found in all sequenced ascomycetes^26,48–50^ and in all sequenced basidiomycetes except in *Coprinopsis cinerea*^40^, whose genome also lacks *FTR1* homologs, but has a *sid1/sidA* gene involved in the biosynthesis of an iron-chelating siderophore involved in iron acquisition under conditions of low soil iron availability^29^.

In several fungal species, at least one of the *Fet3* homologues is arranged in the genome with start-to-start codons in a mirrored tandem with the *Ftr1 *homologue^29^. This arrangement suggests a potential common regulation and function of the genes in iron metabolism, as proposed for the *Fet3/Ftr1* homologs of *S. pombe*^34^, *Phanerochaete chrysosporium*^51^ and *Cryptococcus neoformans*^52^. Similar arrangements have been identified in many other fungi such as *Aspergillus fumigatus*^53^, *F. graminearum*^54^, *Ustilago maydis*^55^ and *Laccaria bicolor*^56^. However, such arrangement does not exist in the *R. irregularis* genome, supporting the idea that this fungus lacks a Fet3-type ferroxidase. Nevertheless, we have found that *RiFTR1* is localized adjacent to the copper transporter *RiCTR2*, whose expression was also observed to be induced in the IRM^57^. This suggests a potential connection between Fe metabolism and copper metabolism in this fungus, as demonstrated in other organisms^58,59^. Our yeast complementation analyses revealed that RiMCO1 and RiMCO3, the two *R. irregularis* MCOs displaying an Fe-regulated expression pattern and the functional domains typical of ferroxidases, are able to bind to yeast Ftr1 protein in the Δ*fet3* mutant forming a complex for the uptake of Fe. However, our complementation analyses in the triple yeast mutant lacking the ferroxidase Fet3 and both the low- and high-affinity Fe uptake systems indicated that RiFTR1 is capable of transporting Fe under Fe-limiting conditions without the help of a ferroxidase partner. Thus, the mechanism of Fe transport by RiFTR1 appears to resemble that of the plant iron-regulated transporter1 (IRT) system, capable of partially restoring the impaired growth of the ∆*fet3*∆*fet4 *yeast mutant under Fe-limited conditions^60,61^. While such mechanism has not been reported in fungi to date, the absence of Fet3-ferroxidases in the *R. irregularis* genome and the lack of correlation between the expression of *RiFTR1* and any *RiMCO* seem to support this possibility. These data suggest that the reductive Fe uptake pathway evolved by AM fungi may be more similar to the reduction strategy of plants than to that of fungi, although AM fungi other than *R. irregularis* should also be investigated.

Higher plants have evolved two different strategies to acquire Fe from soil: a reduction strategy (Strategy I) employed by dicots and non-graminaceous monocots, and a chelation strategy (Strategy II) used by graminaceous species^62^. In the reduction strategy, once Fe is solubilized by the action of a H^+^-ATPase, Fe(III) is reduced to the more soluble form Fe(II), which is subsequently transported into the root epidermal cells via high-affinity iron transporters from the Zrt/Irt-like protein (ZIP) family. Therefore, our data suggest that Fe transport across the *R. irregularis* plasma membrane through the Fe permease RiFTR1 occurs in the form of Fe(II) and that does not require the concerted action of a ferroxidase, as described in fungi that employ a reductive Fe uptake pathway^63^. Given that AM fungi facilitated the initial colonization of land by plants, providing them with inorganic nutrients and moisture from the substrate in exchange for carbon compounds^64^, and considering that roots evolved after the transition to land^65^, along with more than 400 million years of coevolution between AM fungi and plants^66^, it is not surprising that AM fungi and plants employ the same Fe uptake strategy to take up Fe from the soil solution. In fact, it could be hypothesized that plants exploited the reductive Fe uptake strategy previously evolved by AM fungi.

The discovery that the Fe uptake mechanism in *R. irregularis* resembles that of plants rather than the traditional fungal pathway challenges the conventional understanding of fungal Fe uptake and highlights the evolutionary adaptations of mycorrhizal fungi that align them more closely with plant systems. The absence of ferroxidase, an enzyme typically involved in fungal Fe uptake through the oxidation and transport of Fe, indicates a shift towards a more direct and efficient method of Fe acquisition. Despite Fe being one of the most abundant metals in the Earth’s crust, it is not readily bioavailable. The evolution of Fe capture from the Earth’s crust and early soils was suggested to be a crucial step in the terrestrialization of early unicellular streptophytes, which ultimately gave rise to land plants^67^. The plant-like Fe uptake mechanism in mycorrhizal fungi likely reflects the fungi’s role in assisting plants with nutrient acquisition, such as iron, in nutrient-poor soils, a critical factor during the early colonization of early terrestrial environments.

Despite the fact that RiMCO1 and RiMCO3 are not required for the Fe high-affinity uptake system of *R. irregularis*, as demonstrated by growth complementation, we have shown that RiMCO1 and RiMCO3 function as ferroxidases. However, Fe(II) might not be the only or even the preferred substrate for these enzymes. In fact, since these two MCOs share the highest identity with the fungal ferroxidases/laccases, it cannot be excluded that RiMCO1 and RiMCO3 could also exhibit laccase activity and oxidize other substrates. Ferroxidases-lacasses were proposed as hybrid enzymes with dual catalytic properties between laccase and ferroxidase and have been described in different fungi^68–70^. However, their physiological functions have not been sufficiently explored. In the opportunistic fungal pathogen *C. neoformans*, the ferroxidase activity of the ferroxidase-laccase protein has been associated to a virulence factor^68–71^. Unlike fungi different from AM fungi, plants possess a multiplicity of ferroxidases that are not central players in Fe uptake but play different roles in various organs and in response to different nutritional constrains, directly or indirectly related to Fe^58,72,73^. The findings that RiMCO1 and RiMCO3 respond to Fe status and display ferroxidase activity suggest that they might be involved in processes that require a switch from Fe (II) to Fe (III). Moreover, the *RiMCO4 *gene was found to be Fe-regulated, suggesting that it could also have a Fe-related role, perhaps by controlling its oxidation stage and protecting the fungus against the oxidative stress caused by Fe toxicity^74^. Unveiling the physiological roles of the different MCOs identified in the genome of *R. irregularis* requires further studies, including the biochemical characterization of their encoded proteins and additional expression analyses in plants grown in natural soils. This approach will help to address potential limitations associated with ERM grown in root organ cultures on Petri dishes.

Root colonization by *R. irregularis* upon *RiFTR1* overexpression in *M. truncatula* was enhanced when morphologically analyzed, but this increase was not statistically supported by *RiTEF* expression but of *MtPT4*. This suggests that such an increase in colonization rather reflects the higher number and activity of arbuscules, while the measured hyphae might have been septated and not alive. These results, along with RNA-Seq data showing that *RiFTR1 *is expressed in the arbuscules formed in root cortical cells, suggest the importance of RiFTR1 for AM symbiosis, and in particular for arbuscule functioning. These data align with observations in various plant-microbe mutualistic interactions^75–77^and the biotrophic development of several pathosystems^55,78,79^, where high-affinity Fe transport systems were required and of great importance. Given that the *M. truncatula* roots overexpressed *RiFTR1* under the control of the ubiquitin promoter, it is expected that *RiFTR1* will be expressed in every plant root cell, including the rhizodermis. Therefore, it is likely that these plants might have taken up more Fe from the soil, which in turn had modified the pattern of fungal colonization. Because *RiFTR1* is highly expressed in arbuscule-containing cells under normal symbiotic conditions and arbuscules are postulated as the major site of nutrient exchange between the fungus and the plant, RiFTR1 could play a central role in Fe uptake towards the fungus from the periarbuscular space. This function is likely to be crucial to meet the Fe demand of the fungus for the successful development or function of arbuscules being Fe an essential cofactor of several enzymes involved in critical metabolic processes^46,80^. This hypothesis is supported by the observed increase in the gene expression of the *M. truncatula* symbiotic phosphate transporter MtPT4 in roots overexpressing *RiFTR1*.

In conclusion, the findings of this study provide valuable insights into the functionality of the *R. irregularis* Fe transporter RiFTR1. Contrary to previous assumptions, our results clearly demonstrate that RiFTR1 operates independently and does not necessitate a ferroxidase partner for its function. Moreover, this work has provided insights into the role of Fe in AM associations, showing a positive role in root colonization. These findings enhance our understanding of the intricate interplay between mycorrhizal fungi and plants.

## Materials and methods

### Identification of MCO genes in the *R. irregularis* genome and sequence analyses

Amino acid sequences of fungal MCOs were retrieved from the freely accessible transport database TCDB^81^. These sequences were used to search for orthologous sequences in the filtered model dataset of *R. irregularis *on the JGI website^82,83^ using Basic Local Alignment Search Tool (BLAST) algorithm^84^ via a protein BLAST. A second search was performed via a keyword search directly using “multicopper oxidase”, “laccase”, “ascorbate oxidase”, “ferroxidase”, “bilirubin oxidase” and “Fet3” as keywords. Since some of the fungal reference proteins were phylogenetically distant from *R. irregularis*, manually curated *L. bicolor*^85^, *Tuber melanosporum*^86^ and *Rhizopus oryzae*^87^ databases were used to look for additional orthologous sequences in the filtered model dataset of *R. irregularis*. This was also done via a BLASTp, run with the standard program settings. Finally, a keyword search for putative multicopper oxidases was performed on the functional annotation collection from Lin et al.^88^. Sequences were finally compared to the most recent version delivered sequences of *R. irregularis*via BLASTp and keyword search for putative multicopper oxidases^89–95^.

Predictions of putative transmembrane domains were made using the TMHMM Server v.2.0^96^and the TOPCONS server^97^and the presence of the conserved domains was analyzed using SMART software^98,99^.

Full-length amino acid sequences were aligned with the orthologous sequences of a number of fungi representatives of distinct taxonomic groups by ClustalW (Version 2.1^100,101^) with default setting. Alignments were imported into the Molecular Evolutionary Genetics Analysis (MEGA) package version 11^102^. Phylogenetic analyses were conducted by the Neighbor-Joining (NJ) method, implemented in MEGA, with a pair-wise deletion of gaps and the Poisson model for distance calculation. Bootstrap analyses were carried out with 1000 replicates. The evolutionary tree was drawn to scale.

### Biological materials and growth conditions

*Rhizophagus irregularis* monoxenic cultures were established as described by St-Arnaud et al.^103^, with some modifications. Briefly, clone DC2 of carrot (*Daucus carota* L.) Ri-T DNA transformed roots were cultured with the AM fungus *R. irregularis *Schenck and Smith DAOM 197198 in two-compartment Petri dishes. Cultures were initiated in one compartment of each plate containing M medium^104^ by placing several non-mycorrhizal carrot root segments and a piece of fungal inoculum containing ERM, fragments of mycorrhizal roots and spores. Fungal hyphae and roots were allowed to grow over to the other compartment containing the same M medium. Plates were incubated in the dark at 24 °C for 7–8 weeks until the second compartment was profusely colonized by the fungus and the roots. Then, the older compartment was removed and refilled with liquid M medium without sucrose (M-C medium) containing different Fe concentrations: 0.045 mM (control), 4.5 mM or 45 mM EDTA Fe(III) sodium salt. Fungal hyphae, but not roots, were allowed to grow over to this compartment (hyphal compartment). Plates were incubated in the dark at 24 °C for 2–3 additional weeks. To induce Fe-deficient conditions, monoxenic cultures were initiated in plates containing solid M medium without Fe and incubated in the dark for 7–8 weeks as above. Later, fungal hyphae grown (for 2–3 weeks) in liquid M-C medium without Fe were exposed 3 days to 0.5 mM ferrozine (Sigma). This was done by replacing the liquid medium by 20 ml of a freshly prepared liquid M-C medium without Fe and supplemented with 0.5 mM ferrozine, which was prepared by adding 0.2 ml of a 0.5 mM stock solution in 50 mM MES pH 6.1 (ferrozine M media). Control plates were prepared as described above, but the M-C media used to refill the hyphal compartment was supplemented with 2 ml 50 mM MES pH 6.1. Plates were incubated in the dark at 24 °C for 3 additional days. ERM from the different hyphal compartments was directly recovered under sterile conditions using a pair of tweezers, washed with sterile water and dried on filter paper. The mycelium was immediately frozen in liquid nitrogen and stored at -80 °C until used.

The *S. cerevisiae* strains used in this study are listed in Table S1. Strains were grown on YPD or complete synthetic medium (CSM), supplemented with appropriate amino acids.

*Medicago truncatula* Gaertn. cv Jemalong A7 seedlings were used for the host induced gene silencing and overexpression experiments. The seeds were originally obtained from Purkiss Seeds (Australia), but since then new seeds have been produced in the Botanical Garden of the Karlsruhe Institute of Technology (KIT).

### Quantification of mycorrhizal colonization

Root fungal structures were stained with trypan blue for phenotypical analysis and quantification of mycorrhizal colonization^105^. Quantification of mycorrhizal structures was carried out according to Trouvelot et al.^106^.

### Nucleic acid extraction and cDNA synthesis

Total fungal RNA from ERM from the different treatments of *R. irregularis* and mycorrhizal carrot roots was extracted using the RNeasy Plant Mini Kit (QIAGEN, Maryland) and from *M. truncatula* roots with the innuPREP Plant RNA Kit (Analitik Jena), following manufacturer´s instructions. DNase treatment was performed using RNA-free DNase Set (QIAGEN, Maryland) following the manufacturer’s instructions. cDNAs were obtained from 1 µg of total DNase-treated RNA in a 20 µl reaction containing 200 units of Super-Script IV Reverse Transcriptase (Invitrogen) and 50 pmol oligo (dT)_20_ (Invitrogen), according to the manufacturer’s instructions.

### RiMCOs gene isolation

The 5´and 3´ends of the *RiMCO* genes were verified by rapid amplification of cDNA ends (RACE) using the SMART RACE cDNA amplification kit (Clontech, Palo Alto, CA, USA), the corresponding gene-specific primer (Table S2) and 1 µg total RNA from ERM grown in control plates. The full-length cDNA of *RiMCO1*,* RiMCO3*,* RiMCO4 and RiMCO6* was obtained by PCR amplification of *R. irregularis* cDNA, using the corresponding primer pairs (Table S2). PCR products were cloned in the pGEM-T easy vector (Promega, Madison, USA).

All plasmids were amplified by transformation of *E. coli* following standard procedures and purified using the Qiagen Miniprep Kit (Qiagen, Maryland, USA). All sequences and constructs were checked by sequencing before further use. Nucleotide sequences were determined by Taq polymerase cycle sequencing using an automated DNA sequencer (ABI Prism 3130xl Genetic Analyzer, Applied Biosystems, Carlsbad, USA).

### Gene expression analyses

Gene expression was studied by real-time RT-PCR using an iQ^TM^5 Multicolor Real-Time PCR Detection System (Bio-Rad). Each 20 µl reaction contained 1 µl of a 1:10 dilution of the cDNA, 200 nM each primer, 10 µl of iQ™ SYBR Green Supermix 2x (Bio-Rad). The PCR program consisted in a 3-min incubation at 95 °C to activate the hot-start recombinant Taq DNA polymerase, followed by 36 cycles of 30 s at 95 °C, 30 s at 58 °C and 30 s at 72 °C, where the fluorescence signal was measured. The specificity of the PCR amplification procedure was checked with a heat-dissociation protocol (from 58 to 95 °C) after the final cycle of the PCR. Fungal transcript levels were standardized to the *R. irregularis* elongation factor 1-alpha gene levels (GenBank Accession No. DQ282611) and plant transcript levels and *RiEF1α* transcript levels *in planta* were normalized to the translation elongation factor 1-alpha of *M. truncatula* gene levels (*MtTEF1α*, GenBank Accession No. XM_013595882). RT-PCR determinations were performed on three independent biological samples from three replicate experiments. Real-time PCR experiments were carried out three times for each biological sample, with the threshold cycle (Ct) determined in triplicate. The relative levels of transcription were calculated using the 2^− ΔΔCT^method^107^, and the standard error was computed from the average of the ΔCT values for each biological sample. Primers used are listed in Table S2.

### Transcriptomic data analysis

RNA-sequencing (RNAseq) data from laser microdissected *Medicago truncatula* cells, containing arbuscules (ARB) or IRM and of ERM grown in monoxenic cultures, were collected from the NCBI Gene Expression Omnibus database GSE99655^43,44^. RNAseq data were analysed. Normalization of the counts was performed using DESeq2 tool of Galaxy (https://usegalaxy.org/).

### Heterologous expression

For heterologous gene expression analyses, the *RiFTR1*, *RiMCO1* and *RiMCO3 *full-length cDNAs were obtained by PCR amplification of the genes cloned in pGEM-T easy vector (Promega, Madison, USA), using the corresponding primer pairs (Table S2). The RiFTR1::pGEM-T plasmid was obtained in a previous work^36^. PCR products were cloned in the part entry vector pYTK001 via *Bsm*BI Golden Gate assembly^108^. All plasmids were amplified by transformation of *E. coli* following standard procedures and purified using the Hi Yield^®^ Plasmid Mini DNA Isolation Kit (SLG^®^). All sequences and constructs were checked by sequencing before further use. For cassette assemblies, the cassette entry vector pYTK095 was used. *RiFTR1* was cloned via *Bsa*I-v2 assembly with the promoter of *CCW12* and the terminator of *PGK1*, and *RiMCO1* and *RiMCO3* with the promoter of *TDH3* and the terminator of *ADH1*. Multicassettes were constructed via *Bsm*BI assembly using the integration vector pYTK096 and single cassettes or the combination of a pair of FTR-MCO cassettes. The full-length cDNAs of *ScFtr1* and *ScFet3* were also amplified and multicassettes were constructed and used as positive controls in the complementation analyses. In total, five multicassettes were built: *RiFTR1*, *RiFTR1-RiMCO1*, *RiFTR1-RiMCO3*, *ScFtr1* and *ScFtr1-ScFet3* muticassettes.

Yeasts were transformed with the corresponding constructs using a lithium acetate-based method^109^, and transformants (Table S1) were selected in CSM medium by auxotrophy to uracil.

For the complementation assay, a specially designed medium was used^110^. To avoid Fe contamination, new plastic or acid-washed glassware was used for all media preparation. Briefly, the assay medium consisted of CSM made with a yeast nitrogen base lacking Fe and copper (Bio-101), supplemented with 1 mM copper sulfate and 50 mM MES pH 6.1. The Fe-limited medium was the assay medium with 100 µM bathophenanthrolinedisulfonate (BPS) (Sigma) (10 mM stock) and different concentrations of ferrous ammonium sulfate. For the plating assay, the medium contained 2% Bacto agar. Stationary yeast cultures with Δ*fet3* background grown in CSM liquid culture were washed with ice-cold distilled water, resuspended in Fe-limited medium supplemented with 200 µM ferrous ammonium sulfate to an OD_600_ of 0.1 and grown to saturation overnight. Stationary yeast cultures with Δ*fet3*Δ*fet4* and Δ*fet3*Δ*fet4*Δ*ftr1* background were grown in CSM liquid culture supplemented with 100 µM ferrous ammonium sulfate to saturation overnight. These cultures were then washed twice and diluted to an optical density at 600 nm of 1. Serial 1:5 dilutions were spotted (5 µl) onto plates containing the assay media supplemented with BPS and different Fe concentrations to determine the lowest Fe concentration at which each mutant strain was able to grow.

### Protein localization analyses

Localization of *R. irregularis* FTR1 protein in *S. cerevisiae* was analyzed by fusion of the mRuby2 protein gene to the C-terminus using an *mRuby2*-Part3b plasmid via Golden Gate assembly^108^. *mRuby2* and *ScFtr1-mRuby2* were used as negative and positive controls, respectively. The Δ*fet3*Δ*fet4* yeast mutant strain was transformed with the resulting multicassettes. Stationary yeast cultures grown in CSM were washed twice with ice-cold distilled water, diluted in Fe-limited medium supplemented with 200 µM ferrous ammonium sulfate and grown for several hours in this medium to an OD_600_ between 0.8 and 1.6. Cells were washed three times and resuspended in water before visualization. The fluorescence signal was visualized with a Zeiss Axio fluorescent microscope. mRuby2 fusion proteins were imaged using a 542–582 nm filter. Image sets were processed and overlapped using ImageJ (http://fiji.sc/Fiji).

### RiFTR1 host induced gene silencing and overexpression

For RiFTR1 silencing via the plant host, two 300 bp sequences of the RiFTR1 cDNA, targeting the regions − 107 to 193 and 684 to 984 with respect to the start codon, were amplified by PCR and recombined in the Gateway binary RNAi vector pK7GWIWG2D(II)-RedRoot (https://www.psb.ugent.be/cores/gateway_vectors). For constitutive *RiFTR1* overexpression, its open reading frame was PCR amplified from *R. irregularis* cDNA, cloned into pENTR/D-TOPO vector and recombined in the plant expression vector pUBIcGFP-DR^111^. The PCR primers used are listed in Table S2.

The resulting RiFTR1 RNAi (RNAi-1 and RNAi2) and overexpression (RiFTR1-OE) constructs were used to generate transgenic *M*. *truncatula* hairy roots via *Agrobacterium rhizogenes* transformation, as previously described^14,112^. Empty vectors were used as controls. After transformation, plants were transferred into 50 ml Falcon tubes containing sterile sand with *R. irregularis* DAOM197198 previously propagated in monoxenic cultures (1 plate inoculum containing spores, ERM and mycorrhizal roots for 100 ml substrate). To prevent desiccation, plants were covered with sun-transparent bags (Sigma-Aldrich). Plants were incubated at 24/20°C (16 h light/8 h darkness) and watered twice a week with 5 ml of a half-strength low Pi (20 µM) Long Ashton nutrient solution^113^. Transgenic roots were harvested 5 weeks after inoculation with *R. irregularis*. Roots were harvested, used for RNA extraction and stained with trypan blue to visualize fungal structures, as described above.

### Statistical analyses

Statgraphics Centurion XVI software was used for the statistical analysis of the means and standard deviation determinations. Data of each treatment were first subjected to the Shapiro–Wilk test for normality. ANOVA, followed by a Fisher’s LSD test (*p* < 0.05) when possible, was used for the comparison of two treatments based on at least 3 biological replicates for each treatment (*n* ≥ 3). For data comparison of more than two groups and showing no normal distribution, the Kruskal–Wallis test was carried out and the significance calculated according to the *post hoc* Dunn’s test^114^. If data were showing a normal distribution an ANOVA and a Duncan *post hoc* test were carried out. Significant differences were shown with different letters (*p* < 0.05).

## Electronic supplementary material

Below is the link to the electronic supplementary material.

Supplementary Material 1

## Data Availability

The original contributions presented in the study are included in the article/Supplementary Material. Further inquiries can be directed to the corresponding author.
