# Supplementary material for: The Rhizophagus irregularis permease RiFTR1 functions without a ferroxidase partner for reductive iron transport
Source: Sci Rep. 2025 Feb 18;15:5840. doi: 10.1038/s41598-025-88416-3 (PMC11836134; doi:10.1038/s41598-025-88416-3)
Supplement: Supplementary file 1 — Supplementary Material 1 [file 41598_2025_88416_MOESM1_ESM.pdf]

Scientific Reports

The *Rhizophagus irregularis* permease RiFTR1 functions without a ferroxidase partner for reductive iron transport

Elisabeth Tamayo[1,2,*], Víctor Manuel López-Lorca[1], Chaeeun Shim[2,3], Olga López-Castillo[1], Araceli G. Castillo[4], Natalia Requena[5], J. Philipp Benz[2], Nuria Ferrol[1].

[1]Departamento de Microbiología del Suelo y Sistemas Simbióticos, Estación Experimental del Zaidín, CSIC, Granada, Spain.

[2]Holzforschung München, TUM School of Life Sciences, Technische Universität München, Freising, Germany.

[3]Department of Biochemical Engineering, Bernard Katz Building, University College London, London, United Kingdom.

[4]Instituto de Hortofruticultura Subtropical y Mediterránea "La Mayora", Universidad de Málaga-CSIC (IHSM, UMA-CSIC), Málaga, Spain.

[5]Molecular Phytopathology, Botanical Institute, Karlsruhe Institute of Technology (KIT), Karlsruhe, Germany.

*Corresponding author: tamayo@hfm.tum.de

Supplementary Tables

Table S1. ***S. cerevisiae*** **strains used in this work.**

Strain	Relevant genotype	Reference
YPH252	*MATα ura3-52 lys2-801 ade2-101 trp1-Δ1 his3-Δ200 leu2-Δ1*	[1]
42B *ftr1Δ-1*	As YPH252 but *ftr1Δ::TRP1*	[2]
YPHfa	As YPH252 but *fet3Δ::TRP1*	[2]
DDY4	*MAT*a *ura3 can1 trp1 his3 leu2 fet3-2Δ::HIS3 fet4-1Δ::LEU2*	[3]
DEY1530	*MATα ade2 lys2 ura3 leu2 his3 trp1 fet3Δ::HIS3 fet4Δ::LEU2 ftr1Δ::TRP1*	[4]
Δfet3Δfet4 ScFtr1-Fet3	DDY4 transformed with *ScFtr1-Fet3*	This work
Δfet3Δfet4 RiFTR1	DDY4 transformed with *RiFTR1*	This work
Δfet3Δfet4 RiFTR1-MCO1	DDY4 transformed with *RiFTR1-MCO1*	This work
Δfet3Δfet4 RiFTR1-MCO3	DDY4 transformed with *RiFTR1-MCO3*	This work
Δfet3Δfet4Δftr1 ScFtr1-Fet3	DEY1530 transformed with *ScFtr1-Fet3*	This work
Δfet3Δfet4Δftr1 RiFTR1	DEY1530 transformed with *RiFTR1*	This work
Δfet3Δfet4Δftr1 RiFTR1-MCO1	DEY1530 transformed with *RiFTR1-MCO1*	This work
Δfet3Δfet4Δftr1 RiFTR1-MCO3	DEY1530 transformed with *RiFTR1-MCO3*	This work

Table S2. Oligonucleotides used in this study.

Primer	Sequence 5' - 3'	Application
GiFETa.rR	TGCAGGTCCAGGAATTTGTTGAGG	5’ RACE of *RiMCO1*
FETc.rF	ACTTTAGCCCCGGATGGATTTACTCG	3’ RACE of *RiMCO3*
FETc.rR	GAGATTGTCCTGCCACCCCGTCAA	5’ RACE of *RiMCO3*
FETd.rF	GTGTCATCAACACAAGCGCGGAAG	3’ RACE of *RiMCO4*
FETf.rF	TGAAACACAACGTGCGGATGGAC	3’ RACE of *RiMCO6*
FETf.fR	CCAATAGGTTCCAGGATGTGTAGCTTCG	5’ RACE of *RiMCO6*
qRiFTR1F	AGGATCGCATAGGATGTCAA	Real-time PCR of *RiFTR1*
qRiFTR1R	AGAAAAGACCAGCGGCAAC	Real-time PCR of *RiFTR1*
GiFETaF	CTCAACCAGTTGGTCCGTATTT	Real-time PCR of *RiMCO1*
GiFETaR	TACCCATTTCAACATGCCACTC	Real-time PCR of *RiMCO1*
FETb.qF	CATTTCATATGCACGGACAC	Real-time PCR of *RiMCO2*
FETb.qR	CCAGGAACGGTCACATTATC	Real-time PCR of *RiMCO2*
RiFETc.qF	GCATTTGCAATCTGGTCTTT	Real-time PCR of *RiMCO3*
RiFETc.qR	TACACAAGCTTTTCGGCATT	Real-time PCR of *RiMCO3*
RiFETd.qF	CCATTCCCATCATGTCAAAT	Real-time PCR of *RiMCO4*
RiFETd.qR	CCTTGTTTGTTTTCCCTCCT	Real-time PCR of *RiMCO4*
FETe.qF	CCGATCAACATGGTGCCTACT	Real-time PCR of *RiMCO5*
FETe.qR	AGCGTCCTGAAGTGGTAAGAGA	Real-time PCR of *RiMCO5*
FETf.qF	TCAATCCCCTCAGAATCACCAGA	Real-time PCR of *RiMCO6*
FETf.qR	GGTTTTGTCGTTTCAGGTTGTG	Real-time PCR of *RiMCO6*
RiFETg.qF	ACAGGGCAGAATTGTTTCAA	Real-time PCR of *RiMCO7*
RiFETg.qR	TTTCTTGACGCTTTTCGAGT	Real-time PCR of *RiMCO7*
RiFETh.qF	AAAAAGGGCAACAATCACAC	Real-time PCR of *RiMCO8*
RiFETh.qR	ATATACCCCCGGTTCAAAAT	Real-time PCR of *RiMCO8*
RiFETi.qF	CTGCAGAAATGCAAGAGACC	Real-time PCR of *RiMCO9*
RiFETi.qR	CATATGCACGGACATTTTGA	Real-time PCR of *RiMCO9*
qRiEF1αF	GCTATTTTGATCATTGCCGCC	Real-time PCR of *RiEF1α*
qRiEF1αR	TCATTAAAACGTTCTTCCGACC	Real-time PCR of *RiEF1α*

MtPT4-qPCR-F	GTGCGTTCGGGATACAATACT	Real-time PCR of *MtPT4*
MtPT4-qPCR-R	GAGCCCTGTCATTTGGTGTT	Real-time PCR of *MtPT4*
MtTEF1a-qPCR-F	TACTCTTGGAGTGAAGCAGATG	Real-time PCR of *MtTEF1α*
MtTEF1a-qPCR-R	GTCAAGAGCCTCAAGGAGAG	Real-time PCR of *MtTEF1α*
RiFETa_s.fF	ATGGGGAATATTAAAAGAAT	Cloning of *RiMCO1* CDS
FETa.fR	CTATACTTTTATTCTTTTCATATCAGTATT	Cloning of *RiMCO1* CDS
FETc.fF	ATGATTTTTTATAAATTATTTGTTCATCTT	Cloning of *RiMCO3* CDS
RiFETc_l.fR	TTAATATTTCGTATTCAGTTTACATAAATC	Cloning of *RiMCO3* CDS
FETd.fF2	ATGAGAATAACTTCGATAATAGTAT	Cloning of *RiMCO4* CDS
FETd.fR2	CTAATAAGCACTTCCACGTAT	Cloning of *RiMCO4* CDS
FETf.fF	ATGAGAGGTGAAAAGAAATTTC	Cloning of *RiMCO6* CDS
FETf.fR	TTAATTATTATTCCAAATATTTTTGATTGC	Cloning of *RiMCO6* CDS
ScFet3_Fw1	GACGTCTCCtcggTCTCATATGACTAACGCTTTGCTCTCTATAGCCG	Part3a-*ScFet3* plasmid
ScFet3_Rv1	GACGTCTCCGTtTCCGCTTGTGCTAGCGAG	Part3a-*ScFet3* plasmid
ScFet3_ Fw2	GACGTCTCCAaACGCACACGTTTAATTGGACC	Part3a-*ScFet3* plasmid
ScFet3_ Rv2	GACGTCTCCGAGtCCGTGGAAATGCATAGAAGTATTG	Part3a-*ScFet3* plasmid
ScFet3_ Fw3	GACGTCTCCaCTCTTCCAAAACGGAACCGC	Part3a-*ScFet3* plasmid
ScFet3_ Rv3	GACGTCTCCggtcTCAAGAACCGAAGAACCGTTTGGCTTTAGTTAAAAATTG	Part3a-*ScFet3* plasmid
ScFtr1_Fw1	GACGTCTCGtcggTCTCATATGCCTAACAAAGTGTTTAACGTGGCC	Part3a-*ScFtr1* plasmid
ScFtr1_Rv1	GACGTCTCGGAgGAGGAATGACTGGTAGTTTGCTC	Part3a-*ScFtr1* plasmid
ScFtr1_Fw2	GACGTCTCCCcTCTCAAAACGTCGCCACAGAC	Part3a-*ScFtr1* plasmid
ScFtr1_Rv2	GACGTCTCCggtcTCAAGAACCAAGAGAGTCGGCTTTAACGTGGAG	Part3a-*ScFtr1* plasmid
FETa_short Fw	GACGTCTCCTCGGTCTCATATGGGGAATATTAAAAGAATTTTTGCTCTCATA ATAACC	Part3a-*RiMCO1* plasmid
FETa_short Rv	GACGTCTCCGGTCTCAAGAACCTACTTTTATTCTTTTCATATCAGTATTAAAT AGTCTATTCCTTC	Part3a-*RiMCO1* plasmid
FETa_short Fw2	GACGTCTCCACTCATAAGTGGTGCCGGTCG	Part3a-*RiMCO1* plasmid
FETa_short Rv2	GACGTCTCCGAGTCCAGAATCCGGAATAGGATTACG	Part3a-*RiMCO1* plasmid
FETc_long Fw	GACGTCTCGTCGGTCTCATATGATTTTTTATAAATTATTTGTTCATCTTTTTTGCG	Part3a-*RiMCO3* plasmid

FETc_long Rv	GACGTCTCCGGTCTCAAGAACCATATTTCGTATTCAGTTTACATAAATCAACCCA TTC	Part3a-*RiMCO3* plasmid
FETc_long Fw2	GACGTCTCCGTGACCACCCGTTTCACATGC	Part3a-*RiMCO3* plasmid
FETc_long Rv2	GACGTCTCGTCACCTTCGTCAGTATTGATAAAAGTAATATCAACTAC	Part3a-*RiMCO3* plasmid
FTR1 Fw	GACGTCTCCTCGGTCTCATATGGTTTATTTATTCGATGTTCCCGCTTATTTC	Part3a-*RiFTR1* plasmid
FTR1 Rv	GACGTCTCCGGTCTCAAGAACCAGCTGCATTCACAATAGCCGC	Part3a-*RiFTR1* plasmid
RiFTR1.TOPO.F	CACCATGGTTTATTTATTCGATG	Overexpression of *RiFTR1* in *Medicago truncatula*
RiFTR1.TOPO.R	TTAAGCTGCATTCACAATAGCAG	Overexpression of *RiFTR1* in *Medicago truncatula*

Table S3. Protein accession number, predicted nucleotide and amino acid sequence length, introns and transmembrane domains of the *Rhizophagus irregularis* MCOs.

Name	NCBI ID	Nucleotide sequence length (bp)	Amino acid sequence length (aa)	Predicted intron number	Predicted transmembrane domains
RiMCO1	PKC08867	2731	593	9	1
RiMCO2	PKC01889	2188	605	5	0
RiMCO3	POG56303	2836	546•	11	1
RiMCO4	GBC34208	2585	623	9	1
RiMCO5	EXX55101	2159	608	4	1
RiMCO6	EXX61193	2739	708	7	1
RiMCO7	EXX71299	2614	621	9	0
RiMCO8	PKC16282	2593	558	12	3
RiMCO9	PKY12290	2670	539	11	1

•NCBI sequence is shorter. Sequence was experimentally determined by using RACE technology.

Supplementary Figures

A

MCO	RiMCO1	RiMCO2	RiMCO3	RiMCO4	RiMCO5	RiMCO6	RiMCO7	RiMCO8	RiMCO9
RiMCO1		43	38	44	46	25	41	34	38
RiMCO2			34	42	64	26	40	32	34
RiMCO3				39	37	29	35	64	76
RiMCO4					43	26	82	33	37
RiMCO5						27	40	34	36
RiMCO6							24	26	28
RiMCO7								31	34
RiMCO8									61
RiMCO9									

B

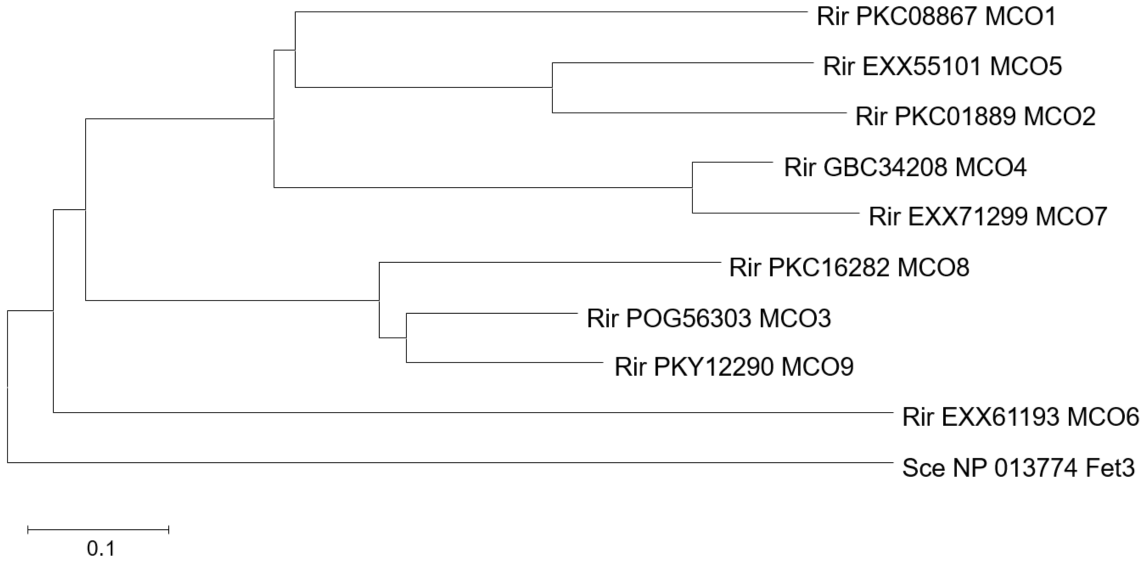

Figure S1. A. Percent sequence identity matrix of putative amino acid sequences of MCOs in *Rhizophagus irregularis*. B. Neighbor-Joining tree of the deduced amino acid sequences of the *R. irregularis* MCOs. The *S. cerevisiae* Fet3 sequence was used as outgroup. Rir, *R. irregularis*; Sce, *S. cerevisiae*. Protein NCBI identification numbers are indicated.

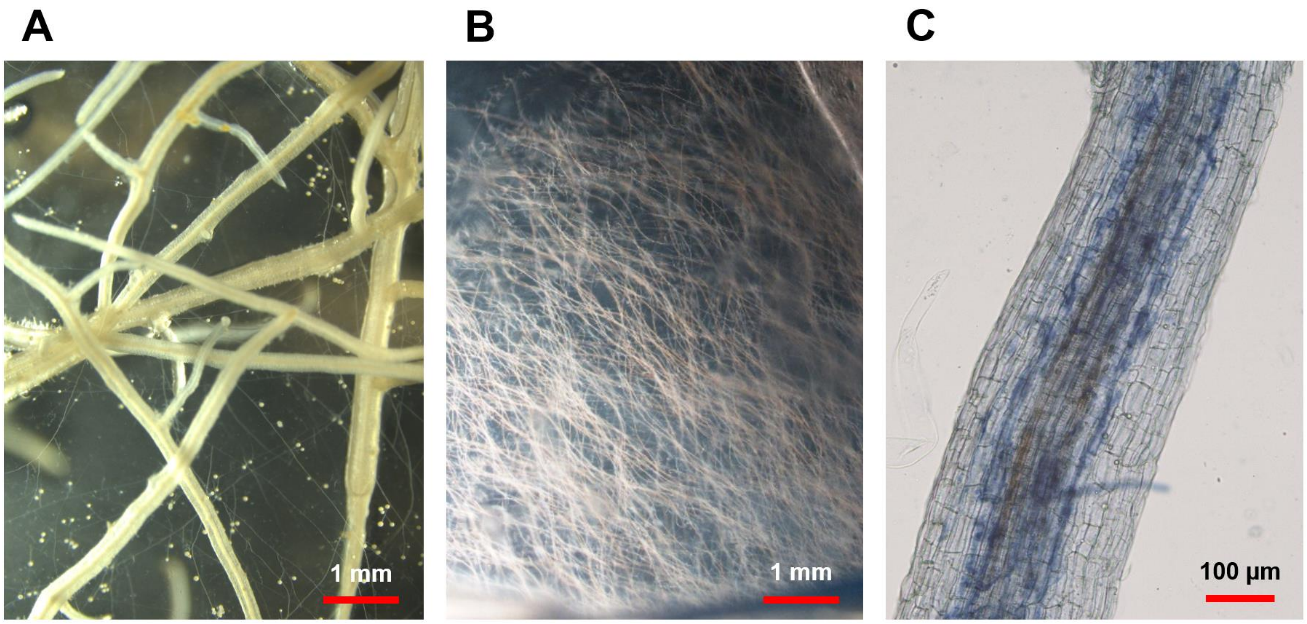

Figure S2. Mycorrhizal carrot (*Daucus carota* L.) roots and extraradical mycelium (ERM) of *Rhizophagus irregularis* grown monoxenically in bi-compartmented Petri dishes. A. View of the root compartment. **B.** View of the hyphal compartment with ERM. **C.** Detail of a mycorrhizal root segment after staining with trypan blue. The arbuscules can be seen colored in blue.

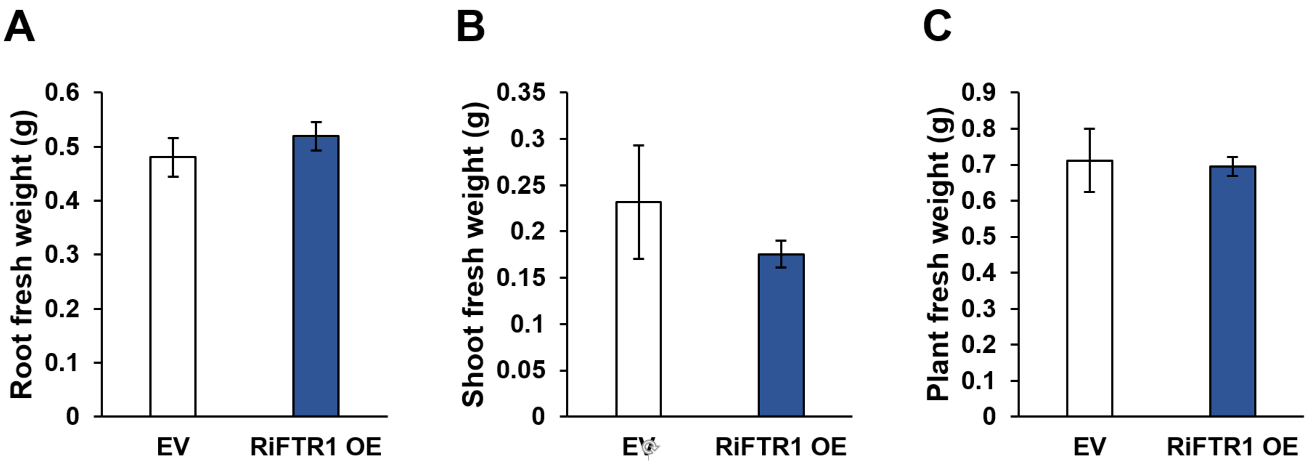

Figure S3. Effect in plant weight of the overexpression of *RiFTR1* in mycorrhizal *Medicago truncatula*. Plant growth parameters (root fresh weight **(A)**, shoot fresh weight **(B)** and total plant fresh weight **(C)**) were measured at the end of the experiment of ectopic expression of *RiFTR1* in mycorrhizal roots of *M. truncatula* composite plants transformed with and empty vector (EV) compared with *RiFTR1*-expressing plants (RiFTR1 OE). Statistical significance is indicated with asterisks (**p* > 0.05). Data are means +/- standard errors (n=4).

Supplementary references

1. Sikorski, R.S., Hieter, P. A system of shuttle vectors and yeast host strains designed for efficient manipulation of DNA in *Saccharomyces cerevisiae*. Genetics 122(1), 19-27. doi: 10.1093/genetics/122.1.19 (1989).

2. Stearman, R., Yuan, D.S., Yamaguchi-iwai, Y., Klausner, R.D., Dancis, A. A permease-oxidase complex involved in high-affinity iron uptake in yeast. Science 271, 1552–1557. doi: 10.1126/science.271.5255.1552 (1996).

3. Dix, D.R., Bridgham, J.T., Broderius, M.A., Byersdorfer, C.A., Eide, D.J. The *FET4* gene encodes the low affinity Fe(II) transport protein of *Saccharomyces cerevisiae*. J. Biol. Chem. 269(42), 26092-9 (1994).

4. Spizzo, T., Byersdorfer, C., Duesterhoeft, S., Eide, D. The yeast *FET5* gene encodes a *FET3*-related multicopper oxidase implicated in iron transport. Mol. Gen. Genet. 256(5), 547-56. doi: 10.1007/pl00008615 (1997).
